# Potential Antioxidant and Anti-Inflammatory Function of *Gynura procumbens* Polyphenols Ligand

**DOI:** 10.3390/ijms22168716

**Published:** 2021-08-13

**Authors:** Hun Hwan Kim, Sang Eun Ha, Preethi Vetrivel, Pritam Bhagwan Bhosale, Seong Min Kim, Gon Sup Kim

**Affiliations:** Research Institute of Life Science and College of Veterinary Medicine, Gyeongsang National University, 501 Jinju-daero, Jinju 52828, Korea; shark159753@naver.com (H.H.K.); sangdis2@naver.com (S.E.H.); preethivetrivel05@gmail.com (P.V.); shelake.pritam@gmail.com (P.B.B.); ksm4234@naver.com (S.M.K.)

**Keywords:** UF-LC/MS, *Gynura procumbens*, polyphenols, phytochemicals, antioxidant, anti-inflammatory

## Abstract

The antioxidant and anti-inflammatory potentials of polyphenols contained in *Gynura procumbens* (GP) extract were systematically analyzed. Polyphenols in GP were analyzed for nine peaks using high-performance liquid chromatography (HPLC) combined with mass spectrometry (MS), and quantitatively determined through each standard. A total of nine polyphenolic compounds were identified in the samples and their MS data were tabulated. To determine the potential of bioactive ingredients targeting DPPH and COX-2, we analyzed them by ultrafiltration combined with LC. The results identified the major compounds exhibiting binding affinity for DPPH and COX-2. Caffeic acid, kynurenic acid, and chlorogenic acid showed excellent binding affinity to DPPH and COX-2, suggesting that they can be considered as major active compounds. Additionally, the anti-inflammatory effect of GP was confirmed in vitro. This study will not only be used to provide basic data for the application of GP to the food and pharmaceutical industries, but will also provide information on effective screening methods for other medicinal plants.

## 1. Introduction

It is known that diseases caused by oxidative and inflammatory stress are largely contributed to by prostaglandins and free radicals produced during metabolic processes in the human body [1]. Because such stress contributes to the onset and progression of various chronic diseases, such as cancer, liver disease, and autoimmune diseases [2], research to overcome it continues to this day. Chemically synthesized drugs are most commonly used to treat disease [3], but long-term use causes side effects on the liver, kidneys, and organs in the body [4]. In contrast, polyphenols, which are secondary metabolites of plants, have a long history as drugs due to their distinct aromatic structures, and compared to chemically synthesized drugs, they show reduced or no toxicity [5]. Therefore, interest has increased in the development of plant materials, such as aspirin [6], developed through willow bark, artemisinin [7], which is found in *Artemisia annua* L. and used as a treatment for malaria, and Tamiflu [8], which is synthesized through star anise; and research using plant materials is being actively conducted [9].

Polyphenols are contained in large amounts in plants, and these polyphenols have excellent antioxidant activity as ROS scavengers [10], and are known to strongly inhibit inflammation through the regulation of inflammation-related signaling pathways and inhibition of the release of inflammatory mediators [11]. In addition, polyphenols have antihypertensive effects by dilating blood vessels [12], and they have various physiologically active effects, such as increase in HDL cholesterol levels [13], and anti-sinusitis effects [14]. As such, plant-based materials have been used as a dietary supplements or medicine based on their various activities [15].

The screening of active ingredients in the early stages of new drug development continues to receive a lot of attention and is a field that requires continuous research. Certain enzymes with physiological functions are promising targets for the discovery of physiologically active substances [16], because the binding force between small molecules and target receptors is an important factor in biological activity [17]. Cyclooxygenase-2 (COX-2), which acts as a catalyst for the synthesis and proliferation of PGE2, an inflammatory mediator, is not observed in normal tissues, but is known to be induced and has been detected in inflammatory and tumor tissues [18]. This suggests that inhibition of COX-2 can inhibit the synthesis and proliferation of PGE2, thereby suppressing tumor and inflammatory responses. In addition, the antioxidant activity of DPPH (2,2-dipheny-1-picrylhydrazyl) used in this study can be measured by using the principle that when free radicals are lost, its color disappears. It has been reported that the removal of free radicals protects cell membranes, and prevents diseases such as cardiovascular, arthritis, cancer, and Alzheimer’s [19]. However, studies correlating complex compounds in plants with biological targets or activities are challenging [18], and studies that provide experimental evidence of components and targets are also lacking.

The original name of *Gynura procumbens* is “*Sambungnyawa*”, a plant that also means eternal life. It is also known as a medicinal plant called “*Sambungcho*” and “diabetic herb”. As a plant belonging to the Asteraceae family, it has been used in places such as China, Vietnam, and Thailand [20]. Studies have reported that *Myeongwolcho* has cardioprotective [21], antihyperglycemic [21], anticancer [22], antioxidant [23], and anti-inflammatory [24] properties. Although many studies have been conducted, studies on the mechanism of action of *Gynura procumbens* are lacking.

*Gynura procumbens* has been reported to contain a variety of polyphenols in abundance [25], thereby exhibiting various physiological activities. However, the mechanism for the targeting of DPPH and COX-2 components of *Gynura procumbens* has not yet been elucidated. As such, our study is the first paper to identify the compounds of *Gynura procumbens* and screen the ligands of DPPH and COX-2 through UF-LC-MS analysis using the principle of specific organic compounds and enzymes bind to target receptors. These studies suggest a method for the simultaneous screening of complex compounds in the early stages of drug development, and provide a powerful tool to discover potential ligands for oxidative stress and inflammation from a variety of medicinal plants.

## 2. Results and Discussion

### 2.1. Separation and Characterization of Gynura procumbens Polyphenols

**Figure 1:**
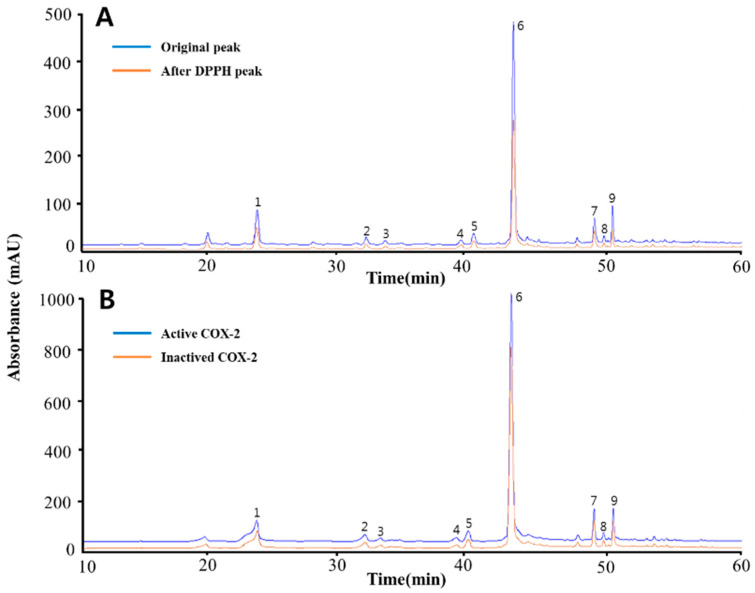
The HPLC chromatograms of the phenolic compounds in *Gynura procumbens.* The blue line in (**A**) is the original chromatogram at the beginning of the *Gynura procumbens*, while the orange line is the chromatogram after reacting with DPPH. The blue line in (**B**) is a chromatogram of the phenolic compound after reacting with activated COX-2, while the orange line is the chromatogram after reacting with inactivated COX-2. The detected compounds at the 284nm wavelength are chlorogenic acid (1), oxypeucedanin (2), isoimperatorin (3), indol-3-carboxylic acid (4), kynurenic acid (5), caffeic acid (6), coumaric acid (7), lutein (8) and luteolin (9).

Quantitative and qualitative analysis of the compounds contained of the *Gynura procumbens* was obtained through HPLC-MS/MS. A total of nine peaks were identified based on HPLC retention time and UV-vis spectrum (Figure 1). Nine phenolic compounds were identified according to the peaks obtained by HPLC chromatograph at a wavelength of 248nm. The nine polyphenolic compounds were identified as chlorogenic acid [26], oxypeucedanin [27], isoimperatorin [27], indol-3-carboxylic acid [28], kynurenic acid [29], caffeic acid [30], coumaric acid [30], lutein [31] and luteolin [32] based on fragmentation patterns. Table 1 provides a description of the mass spectrometry quantitation data based on reference compounds from published sources.

**Figure 2:**
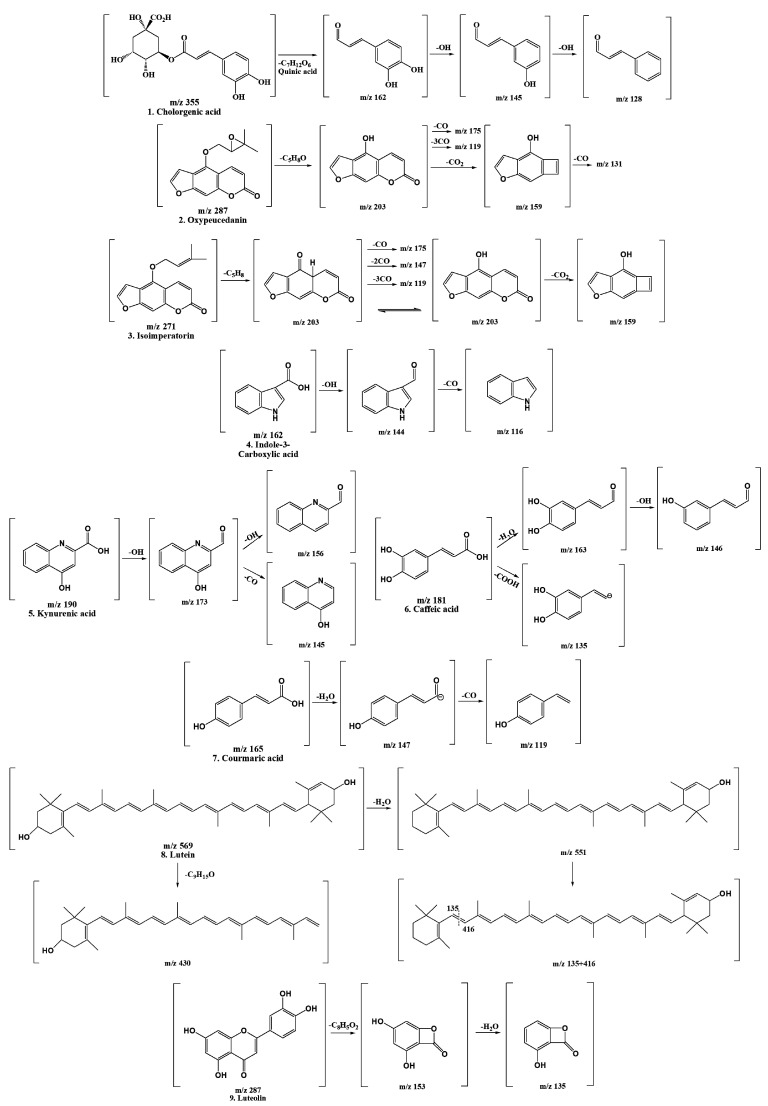
Fragmentation scheme of the polyphenols contained in *Gynura procumbens*.

Our research results support the notion that the bioactive substances contained in natural plant materials can vary depending on climate change and geographic location. This study focused on characterizing the detected polyphenols, and the polyphenols detected based on molecular ion peaks and mass patterns obtained by LC-MS/MS were confirmed in a comparison with the literature data. After that, the retention time with the standard substance and the spectrum UV max value were compared and confirmed once more. Figure 2 shows the result of predicting the cleavage of the compound based on the LC-MS/MS data.

### 2.2. Method Performance, Validation, and Quantification

Quantification of the nine polyphenols detected in *Gynura procumbens* was measured by HPLC-UV chromatograms (284 nm), which matched each standard compound (*n* = 5). A calibration curve obtained from a standard compound consistent with the structural properties described in the previous characterization results was used, as shown in Table 2. Briefly, the determined coefficients (R^2^) were in the range 0.999–1 for all test standards, indicating good linearity. The limits of detection (LOD) were in the range 0.02–0.332 mg/mL, while the limits of quantitation (LOQ) were in the range 0.061–0.996 mg/mL. Table 3 lists the contents of the individual compounds, and they were high in the order of caffeic acid, kynurenic acid, and chlorogenic acid. Among them, indole-3-carboxylic acid and caffeic acid were 6.36 ± 0.01 and 180.69 ± 0.01 μg/mL, respectively, which was about 28 times the content difference.

LODs of 0.029 and 0.134 mg/L, and LOQs of 0.086 and 0.403 mg/L of caffeic acid and cholorogenic acid, which are contained present in high amount in *Gynura procumbens*, which is lower than the previous studies (caffeic acid—LOD (0.08 µg/mL), LOQ (0.27 ug/mL), cholorogenic acid—LOD (0.79 ug/mL), LOQ (2.62 ug/mL)) [33]. Since lower limits of detection (LOD) and limits of quantitation (LOQ) indicate that the precision of the analytical equipment is better, the LOD and LOQ results of this study are considered to have high precision.

### 2.3. Screening for Antioxidants in Gynura procumbens Polyphenolic Compounds

DPPH–HPLC analysis was used to search for potential antioxidant candidates contained in *Gynura procumbens*. In general, DPPH radical scavenging analysis was applied in consideration of the complexity of phytochemicals contained in natural products, and the dependence of various reaction mechanisms in antioxidant analysis. When the free radical contained in the dark purple DPPH (2,2-Diphenyl-1-picrylhdrazyl) reacts with an electron or H atom donor and becomes bonded, it turns yellow, and the antioxidant activity can be measured [34]. According to the LC–MS/MS results, *Gynura procumbens* contains various physiologically active substances, which react with DPPH, and change their content. Figure 1A confirms the change in the compounds that reacted competitively with DPPH through the chromatogram, while Table 3 confirms the reacted amount and reacted rate (%).

Table 3 shows that the radical scavenging activity of these compounds showed a result of reaction to DPPH in a large amount, as did the compounds of higher content. Caffeic acid, known for a long time as an organic compound with antioxidant and anti-inflammatory effects [35], reacted with DPPH at 76.18 ug/mL, and kynurenic and chlorogenic acid at 47.05 and 34.33 ug/mL, respectively, reacted with antioxidation in *Gynura procumbens* extract. It has been confirmed that it is a compound that significantly contributes to its activity. Chlorogenic acid [36], a phenolic ether synthesized by caffeic acid and quinic acid via the phenylpropanoid pathway, and kynurenic acid [37], a tryptophan metabolite, have been reported to provide antioxidant, anti-inflammatory and anti-aging benefits [38,39]. Oxypeucedanin reacted with DPPH in a relatively small amount, but 44.06% of the total content reacted with DPPH, confirming its potential as an antioxidant.

These results indicate that three compounds (caffeic acid, kynurenic acid and chlorogenic acid) act as the main active compounds for the antioxidant activity of *Gynura procumbens*. In addition, compounds with a high reaction rate react more competitively than compounds with a low reaction rate, suggesting that they are more effective in antioxidant activity.

### 2.4. Screening for COX-2 Ligand in Gynura procumbens Polyphenolic Compounds

COX-2 (Cyclooxygenase 2) used in the study oxygenates arachidonic acid, and causes transformation into PGE2, which plays a role in causing fever and pain as a mediator of inflammation in the human body [40]. Therefore, if the ingredients contained in the plant extract act as a ligand for COX-2, it can suppress inflammation in the human body, reducing the conversion of arachidonic acid to PGE_2_.

In general, the compounds contained in the extract are separated, and compared for their activity. However, biocompatible ultrafiltration provides important insights into the ligand receptor binding properties of certain bioactive candidates, while allowing comparative analysis of the compounds contained in the extract [17,41]. In addition, the method of evaluating the potential of the compounds contained in plant extracts using the reacted power of the active compound and the target receptor is an effective research method, because it takes less labor and time than the method of separating and comparing pure compounds.

The chromatogram in Figure 1B shows a distinct difference between before and after reacting to COX-2, which is the result of demonstrating its potential as a COX-2 ligand by specifically reacting to COX-2 by the nine phenolic compounds contained in *Gynura procumbens*. In addition, the peaks of the chromatogram were quantitatively analyzed, and Table 4 indicates the changes before and after reaction as a ligand.

Based on the amount of compounds bound to COX-2, the compounds mainly used for anti-inflammatory response among *Gynura procumbens* extracts were identified, and the compounds effective for anti-inflammatory action were identified through the reaction rate (%).

Overall, the compounds contained in *Gynura procumbens* showed specific binding to COX-2 and thus were considered potential COX-2 ligands. In particular, caffeic acid (43.63 ug/mL), which has been reported to be effective in chronic inflammation including myocardial infarction, arteriosclerosis and blood clots, responded the most [42]. Next, kynurenic acid and chlorogenic acid reacted in large amounts, suggesting that these are mainly used for anti-inflammatory action together with caffeic acid. In addition, indol-3-carboxylic acid (1.66 ug/mL) reacted with COX-2 in a relatively small amount was confirmed to be the most effective compound as 11.06% of the total content was reacted.

In conclusion, the potential of compounds contained in *Gynura procumbens* as COX-2 ligands was confirmed. In addition, the data obtained through this research method can be used as basic data when studying the main and effective compounds that anti-inflammatory effects in other plant materials.

### 2.5. Anti-Inflammatory Effects of Gynura procumbens Polyphenolic Extract (GPE)

Our results indicate that the phenolic compound contained in GPE showed potential as a COX-2 ligand. Therefore, we additionally confirmed the anti-inflammatory effect in vitro.

#### 2.5.1. Cytotoxicity of GPE on RAW 264.7 Cells

**Figure 3:**
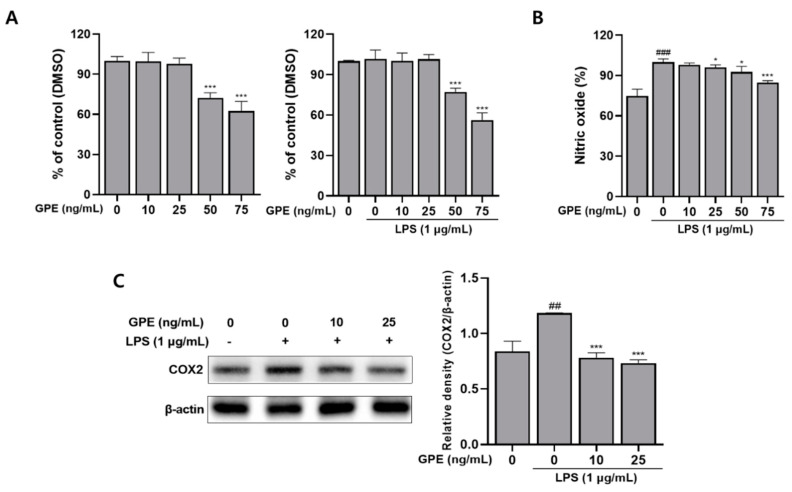
(**A**) Effect of GPE with or without LPS-induced cell viability in RAW264.7 cells. (**B**) Effect of GPE on LPS-induced production of the inflammatory mediators NO. (**C**) Effect of GPE on LPS-induced COX-2 protein expression level in RAW264.7 cell. The results obtained from three independent experiments were expressed as mean ± standard deviation (SD), compared with the control group. ^##^ *p* < 0.005 and ^###^ *p* < 0.001 vs. untreated group; * *p* < 0.05, *** *p* < 0.001 vs. LPS-treated group. GPE; *Gynura procumbens* polyphenolic extract, COX-2; cyclooxygenase-2.

To determine the cytotoxicity of PGE, an MTT assay was used in RAW 264.7 macrophage cells (Figure 3A). RAW 264.7 cells were treated with GPE at various concentrations of (0, 10, 25, 50, and 75) ng/mL with or without 1 µg/mL LPS for 24 h. Figure 3A shows that GPE at (10 and 25) ng/mL exhibited non-toxicity. As a result, those doses were considered to not be cytotoxic to RAW 264.7 cells, and were used for further experiments.

#### 2.5.2. GPE Inhibits LPS-Induced NO Production

The production of NO, the inflammatory mediator, can be inferred from the NaNO_2_ content in the culture medium of cells using the Griess reaction [10]; through this, the effect of inhibiting NO production of GPE in RAW 264.7 cells in which inflammation was induced by LPS was confirmed (Figure 3B). It was confirmed that the treatment of LPS induced a significant increase in NO production, and the treatment of GPE decreased the NO content in a dose-dependent manner. NO is known to regulate the initial stage of migration of inflammatory cells to the inflammatory site [43], and the inhibition of NO production reduces swelling and redness of the infected site by regulating the inflammatory response of macrophages [44]. Therefore, it is suggested that GPE can reduce the swelling and redness of the infected area by controlling the initial stage of inflammation by inhibiting the production of NO.

#### 2.5.3. GPE Inhibits the LPS-Induced Protein Expression of COX-2

The effect of GPE on the protein expression of COX-2 in LPS-induced RAW 264.7 cells was evaluated by Western blot assay. Phosphorylation of MAPK, which performs an important role in the regulation of transcription in the inflammatory process, is regulated by serine and threonine protein kinases. It activates NF- κB, and mediates the expression of LPS-stimulated COX-2 [45]. This COX-2 acts as a catalyst for the conversion of arachidonic acid to prostaglandins, which are the inflammatory mediators [40]. In Figure 3C, LPS-induced RAW 264.7 cells significantly increased the expression of COX-2, compared to the control group not treated with LPS. However, as a result of treatment with GPE, the expression of COX-2 was significantly reduced in LPS-induced RAW 264.7 cells. These results suggest that GPE downregulated the expression of LPS-induced COX-2.

## 3. Materials and Methods

### 3.1. Plant Material

The fresh *Gynura procumbens* was obtained from a well-being village (Chilgok-gun, Gyeongbuk, Korea), a professional farm, and used for the experiment. The leaves of *Gynura procumbens* were washed separately with water, then cut into small pieces, and dried in an oven (55 °C for 72 h). After that, the leaves were stored in a self-sealing polyethylene bags with silica gel at −20 °C, until use.

### 3.2. Reagents and Standards

The recombinant human COX-2 (Cyclooxygenase 2), DPPH (2,2-Diphenyl-1-picrylhdrazyl) and 9 standard compounds (chlorogenic acid, oxypeucedanin, isomperatorin, indol-3-carboxylic acid, kynurenic acid, caffeic acid, lutein, and luteolin) were provided by Sigma-Aldrich Corp. (St. Louis, MO, USA). The purities of all standards were confirmed by HPLC to be >99%. Centrifugal ultrafiltration filters of 30 kDa (YM-30) were purchased from Millipore Co. Ltd. (Bedford, MA, USA). All other chemicals and solvents were of analytical grade, and were obtained from Duksan Pure Chemical Co. Ltd. (Dongdaemun-gu, Seoul, Korea).

### 3.3. Extraction and Purification of Gynura procumbens Polyphenolic Compounds

The isolation of polyphenols from plants was performed using a modified technique [46]. The lyophilized plants (50 g) were extracted in 70% methanol (4 L) for 5 days (d) at room temperature (RT). The mixture was filtered through a filter paper (Whatman qualitative No. 6, 185 mm Ø), and concentrated to 500 mL at reduced pressure at 45 °C, using a rotary evaporator(N-1110, Eyela, Tokyo, Japan) at 100 rpm. The concentrated solution was washed three times with hexane (500 mL) to remove fatty impurities, and ethyl acetate (250 mL) was added to the remaining filtrate three times to extract. Subsequently, after removing moisture with MgSO_4_, the residue was eluted with a silica gel solvent (40 cm × 2.5 cm) and ethyl acetate to remove impurities that had a high polarity. Next, the solvent was concentrated under reduced pressure to obtain a polyphenol mixture powder (0.9 g, 1.8% of the lyophilized plants). The powder was stored at −70 °C, until use.

### 3.4. HPLC and LC-MS/MS Analysis

HPLC and LC-MS/MS was performed on a 1260 series HPLC system (Agilent Technologies, Inc., California, USA) and 3200 QTrap tandem mass system (Sciex LLC) operated in positive ion mode (spray voltage set at −4.5 kV). The solvent used was DW and Acetonitrile containing 0.1% formic acid, a gradient system was used at a flow rate of 0.5 mL/min for analysis, and a Prontosil C18 column (length, 250 mm; inner diameter, 4.6 mm; particle size, 5 µm; Phenomenex Co., Ltd., California, USA,; Biochoff Chromatography) was used. The solvent conditions used in the mobile phases were 0–10 min at 10–15% B, 10–20 min at 20% B, 20–30 min at 25%, 30–40 min at 40%, 40–50 min at 70%, 50–60 min at 95%, and 60–70 min at 95%. The analysis was performed at a wavelength of 284 nm, and temperature of 35 °C. The content of the polyphenolic compound was quantified by the peak area obtained from UV and the standard material.

### 3.5. Quantification of Polyphenolic Compound

Polyphenol samples were quantified in a LC–UV chromatogram with 9 selected standards. Quantification of polyphenolic compound detected in sample of *Gynura procumbens* was conducted using HPLC at 284 nm. The quantification performance was validated in terms of linearity, limit of detection (LOD), and limit of quantitation (LOQ). A calibration curve was established for each of the standards using five concentration levels (*n* = 5; (62.5, 125, 250, 500, and 1000) μg/mL), and the polyphenol content was measured in terms of peak area ratios with the analyte vs. analyte concentrations using a 1/x (x, concentration) weighted linear regression (*n* = 5). Plant polyphenolic compounds are routinely quantifiable using standard curves of structurally related compounds, but we quantified using standards that matched each compound.

### 3.6. DPPH–HPLC Analysis for the Screening of the Main Antioxidants in Polyphenolic Compound

The method adopted for screening the main antioxidant compounds among the polyphenols in the *Gynura procumbens* extract was obtained through a few modifications to the technique [47]. Briefly, polyphenolic compounds (1000 μg/mL) and 5 mg/mL DPPH reagent were mixed at a ratio of 1:1 (*v*:*v*), and then reacted at RT for 15 min. After that, the mixture was filtered through a 0.45 μm filter, and analyzed via HPLC. As a control, methanol was used instead of DPPH reagent. By analyzing the chromatographic peak values and the standard curve values of the samples and control reacted with DPPH, the content of the compounds reacted with DPPH could be obtained. This allowed the major antioxidants from *Gynura procumbens* phenolic compounds and their information to be screened.

### 3.7. Identification of the Potential COX-2 Ligands of Polyphenolic Compound Using UF–HPLC

The plant polyphenols were isolated using the technique performed in a previous reported study with a few modifications [1]. Briefly, 100 μL of the polyphenolic extract was reacted with 20 μL of COX-2 (2 U) for 30 min at 37 °C. On the other hand, the negative control was reacted in the same condition as COX-2, which was deactivated by boiling in a water bath for 15 min. After the reaction was completed, the mixed solution was centrifuged at 10,000 rpm, and ultrafiltered through a 30 KD cutoff ultrafiltration membrane. Subsequently, the free compound was removed by washing three times with 200 μL of NE buffer (pH 7.9, 25 °C) in the filtrate that did not pass through the filter. After that, the remaining compounds were dissolved three times with 80% of ACN, and analyzed through HPLC.

### 3.8. Measurement of Anti-Inflammatory Activity

#### 3.8.1. Cell Culture and Viability Assay

RAW264.7 cells were obtained from the American Type Culture Collection, and cultured in complete DMEM (Gibco, Thermo Fisher Scientific, Inc., Massachusetts, USA) containing 10% heat-inactivated fetal bovine serum (FBS, Gibco, Thermo Fisher Scientific, Inc.), and supplemented with penicillin (100 U/mL) and streptomycin (100 µg/mL; Thermo Fisher Scientific, Inc.). The cells were incubated at 37 °C in a humidified atmosphere containing 5% CO_2_.

RAW264.7 cells were seeded at a density of 1 × 10^4^ cells per well in 96-well plates for 12 h. Next, the cells were treated with GPE of (10, 25, 50, and 75) ng/mL, with or without LPS of 1 µg/mL (Sigma-Aldrich, Merck KGaA, Bulington, USA) for 24 h. After incubation, 10 µL of MTT solution (5 mg/mL) was added to each well, and the cells were incubated for 4 h at 37 °C. The insoluble formazan crystals formed were dissolved in DMSO. Finally, the optical density (OD) value at 450 nm of each well was read with the micro-plate reader (BioTek, Winooski, VT, USA), and each sample was conducted in triplicate.

#### 3.8.2. Measurement of NO by Griess Assay

The nitrite production was measured using the Promega Griess Reagent system (Promega Corporation, Madison, USA), according to the manufacturer’s protocol. RAW264.7 cells were seeded at a density of 1 × 10^4^ cells per well in 96-well plates for 12 h. Next, the cells were treated with GPE of (10, 25, 50, and 75) ng/mL, with or without LPS 1 µg/mL (Sigma-Aldrich, Merck KGaA) for 24 h. After incubation, an equal volume of 100 µL medium, 50 µL sulfanilamides solution, and 50 µL Griess reagent were mixed for 10 min at RT, protected from light. The nitrite concentration was measured by the absorbance at 540 nm.

#### 3.8.3. Determination of Protein Expression by Western Blot Analysis

RAW264.7 cells were seeded at a density of 5 × 10^4^ cells per well in 60 mm plates. The cells were treated with the indicated concentrations of GPE of (10, 25, 50, and 75) ng/mL, with or without LPS 1 µg/mL (Sigma-Aldrich, Merck KGaA) for 24 h. Then, the cells were lysed in RIPA buffer (50 mM Tris-HCl (pH 8.0), 0.5% sodium deoxycholate, 1 mM EDTA, 150 mM NaCl, 0.1 SDS, and 1% NP-40). Protein concentrations were determined using the Pierce bicinchoninic acid protein assay kit (Thermo Fisher Scientific, Inc.), according to the manufacturer’s protocol. Equal amounts of protein (10 µg) were separated via SDS-PAGE on 10% gels, and transferred onto PVDF membranes TE 77 Semi-Dry Transfer Unit (GE Healthcare Life Sciences). The blots were then blocked with 5% bovine serum albumin (BSA, Thermo Fisher Scientific, Inc.) for 1 h 30 min at RT. Membranes were further incubated with primary antibodies (COX-2 (cat. no. 12282S); 1:1000, β-actin (cat. no. 4970S); 1:10,000) for overnight at 4 °C. Primary antibodies of COX-2 and β-actin were purchased from Cell Signaling Technology, Inc.(Massachusetts, USA). The membranes were washed 5 times for 15 min with TBS-T, and membranes were incubated with anti-rabbit (cat. no. A120-101P, Bethyl Laboratory, Inc.) for 3 h at RT. The blots were visualized with an enhanced chemiluminescence kit (Bio-Rad Laboratories, Inc., California, USA). The images were acquired by the ChemiDoc imaging system (Version 6.0, Bio-Rad Laboratories, Inc., California, USA) The β-actin protein was used as a loading control. Western blot images were quantified using the ImageJ 1.50i software (National Institutes of Health), and the experiment was performed in triplicate.

### 3.9. Statistical Analysis

The test measurements were expressed as mean ± standard deviation (M±SD) in triplicate measurements. Statistical analysis was performed using SPSS version 12.0 (SPSS Inc., Chicago, IL, USA), and one-way factorial analysis of variance (ANOVA). Statistical significance was analyzed by Duncan’s multiple range and Student’s test at *p* < 0.05 level, after one-way analysis of variance.

## 4. Conclusions

This study confirmed the action mechanism of the compounds contained in *Gynura procumbens* simultaneously using DPPH–HPLC and bio-affinity UF-HPLC method. Through LC-MS/MS, nine compounds contained in the polyphenol extract of *Gynura procumbens* were identified and quantified, and the reaction amount and reaction rate (%) were confirmed to effectively work with the main compounds used for antioxidant and anti-inflammatory activity. Through this, it was confirmed that three compounds (caffeic acid, kynenic acid, and chlorogenic acid) of the nine compounds contained in *Gynura procumbens* were the main compounds as ligands for DPPH and COX-2, and the potential of other compounds as ligands was also confirmed. Additionally, *Gynura procumbens* extract significantly reduced COX-2 protein expression in LPS-induced RAW 264.7 macrophage cells. To the best of our knowledge, this is the first study to use LC-MS/MS to interpret the compounds of *Gynura procumbens*, and to identify potential ligands through the investigation of antioxidant and anti-inflammatory mechanisms. These results not only provide a powerful tool for quickly discovering potential compounds for antioxidant and anti-inflammatory activity in natural resources, but they can also be used as basic data for the application of *Gynura procumbens* in the near future to the food and pharmaceutical industry.

## Figures and Tables

**Table 1:** The HPLC-MS/MS data of polyphenols from *Gynura procumbens*.

Peak No.	Rt (Min)	Formula	Compound	UV Max	[M+H]^+^	MS/MS
1	23.450	C_16_H_18_O_9_	Chlorogenic acid	330, 322	355	163 (C_9_H_6_O_3_) [M+H-C_7_H_12_O_6_]^+^ 145 (C_9_H_5_O_2_) [M+H-C_7_H_12_O_6_-OH]^+^ 128 (C_8_H_5_O_2_) [M+H-C_8_H_13_O_7_]^+^
2	31.877	C_16_H_14_O_5_	Oxypeucedanin	320, 250	287	203 (C_11_H_6_O_4_) [M+H-C_5_H_8_O]^+^ 175 (C_10_H_6_O_3_) [M+H-C_5_H_8_O-CO]^+^ 159 (C_10_H_6_O_2_) [M+H-C_5_H_8_O-CO_2_]^+^ 131 (C_9_H_6_O) [M+H-C_6_H_8_O_3_-CO]^+^ 119 (C_8_H_6_O) [M+H-C_5_H_8_O-3CO]^+^
3	33.338	C_16_H_14_O_4_	Isoimperatorin	310, 250	271	203 (C_11_H_6_O_4_) [M+H-C_5_H_8_]^+^ 175 (C_10_H_6_O_3_) [M+H-C_5_H_8_-CO]^+^ 159 (C_10_H_6_O_2_) [M+H-C_5_H_8_-CO_2_]^+^ 147 (C_9_H_6_O_2_) [M+H-C_5_H_8_-2CO]^+^ 119 (C_8_H_6_O) [M+H-C_5_H_8_-3CO]^+^
4	39.185	C_12_H_13_NO_2_	Indol-3-Carboxylic acid	280	162	144 (C_12_H_12_NO) [M+H-OH]^+^ 116 (C_11_H_12_N) [M+H-OH-CO]^+^
5	40.185	C_10_H_7_NO_3_	Kynurenic acid	345, 330, 245	190	173(C_10_H_6_NO_2_) [M+H-OH]^+^ 156 (C_10_H_5_NO) [M+H-OH-OH]^+^ 145 (C_9_H_6_NO) [M+H-OH-CO]^+^
6	43.242	C_9_H_8_O_4_	Caffeic acid	320, 285	181	163 (C_9_H_6_O_3_) [M+H-H_2_O]^+^ 146 (C_9_H_5_O_2_) [M+H-H_2_O-OH]^+^ 135 (C_8_H_7_O_2_) [M+H-COOH]^+^ 117 (C_8_H_5_O) [M+H-COOH-H_2_O]^+^
7	49.517	C_9_H_8_O_3_	Coumaric acid	300, 275	165	147 (C_9_H_6_O_2_) [M+H-H_2_O]^+^ 119 (C_8_H_6_O) [M+H-H_2_O-CO]^+^
8	50.243	C_40_H_56_O_2_	Lutein	440, 425	569	551 (C_40_H_54_O) [M+H-H_2_O]^+^ 430 (C_31_H_41_O) [M+H-C_9_H_15_O]^+^ 416 (C_30_H_39_O) [M+H-H_2_O-C_10_H_15_]^+^ 135 (C_10_H_15_) [M+H-H_2_O-C_30_H_39_O]^+^
9	50.895	C_15_H_10_O_6_	Luteolin	350, 268	287	153 (C_7_H_4_O_4_) [M+H-C_8_H_6_O_2_]^+^ 135 (C_7_H_2_O_3_) [M+H-C_8_H_6_O_2_-H_2_O]^+^

Rt: retention time.

**Table 2:** Calibration curves data for the quantification of polyphenolic compounds in *Gynura procumbens*.

Peak No.	Compound	Slopes of Calibration	Correlation Coefficient (r^2^)	LOD (mg/L)	LOQ (mg/L)
1	Chlorogenic acid	14.545	0.9990	0.134	0.403
2	Oxypeucedanin	11.8	1	0.093	0.279
3	Isoimperatorin	7.0775	0.9997	0.273	0.820
4	Indol-3-Carboxylic acid	29.518	0.9993	0.030	0.090
5	Kynurenic acid	3.8206	0.9996	0.332	0.996
6	Caffeic acid	38.079	0.9995	0.029	0.086
7	Coumaric acid	66.712	0.9999	0.020	0.061
8	Lutein	6.5494	0.9996	0.182	0.547
9	Luteolin	18.597	0.9999	0.046	0.139

LOD, limit of detection; LOQ, limit of quantitation.

**Table 3:** Screening of antioxidants in the *Gynura procumbens* polyphenolic compounds.

Peak No.	Compound	Initial Concentration (μg/mL)	Concentration after DPPH Reaction (μg/mL)	Reactive Concentration (μg/mL (%))
1	Chlorogenic acid	85.16 ± 0.06 ^G^	50.84 ± 0.01 ^G^	34.33 ± 0.06 ^G^ (40.31 ± 0.04 ^F^)
2	Oxypeucedanin	13.78 ± 0.19 ^C^	7.70 ± 0.18 ^C^	6.07 ± 0.11 ^C^ (44.06 ± 0.79 ^I^)
3	Isoimperatorin	32.77 ± 0.01 ^E^	21.84 ± 0.03 ^D^	10.93 ± 0.03 ^E^ (33.36 ± 0.09 ^D^)
4	Indol-3-Carboxylic acid	6.36 ± 0.01 ^A^	4.50 ± 0.01 ^A^	1.85 ± 0.01 ^A^ (29.17 ± 0.21 ^C^)
5	Kynurenic acid	107.62 ± 0.13 ^H^	60.57 ± 0.1 ^H^	47.05 ± 0.22 ^H^ (43.72 ± 0.16 ^H^)
6	Caffeic acid	180.69 ± 0.01 ^I^	104.52 ± 0.01 ^I^	76.18 ± 0.02 ^I^ (42.16 ± 0.01 ^G^)
7	Coumaric acid	8.40 ± 0.01 ^B^	5.34 ± 0.01 ^B^	3.06 ± 0.01 ^B^ (36.39 ± 0.12 ^E^)
8	Lutein	30.60 ± 0.06 ^D^	22.97 ± 0.06 ^E^	7.63 ± 0.01 ^D^ (24.93 ± 0.04 ^A^)
9	Luteolin	56.04 ± 0.02 ^F^	40.87 ± 0.02 ^F^	15.16 ± 0.02 ^F^ (27.06 ± 0.03 ^B^)

All values are mean ± SD (*n* = 3). ^A–I^ Means with different superscripts in the same column are significantly different at *p* < 0.05 by Duncan’s multiple range tests.

**Table 4:** Screening of COX-2 ligands of the polyphenol compounds in *Gynura procumbens*.

Peak No.	Compound	With Active COX-2 Concentration (μg/mL)	With Inactive COX-2 Concentration (μg/mL)	Concentration Reacted with COX-2 (μg/mL (%))
1	Chlorogenic acid	241.40 ± 0.02 ^H^	225.67 ± 0.04 ^G^	15.74 ± 0.04 ^G^ (6.97 ± 0.02 ^C^)
2	Oxypeucedanin	59.03 ± 0.01 ^D^	55.22 ± 0.01 ^D^	3.80 ± 0.01 ^E^ (6.88 ± 0.02 ^C^)
3	Isoimperatorin	51.00 ± 0.10 ^C^	47.96 ± 0.02 ^C^	3.03 ± 0.12 ^C^ (6.33 ± 0.25 ^B^)
4	Indol-3-Carboxylic acid	16.65 ± 0.02 ^A^	15.00 ± 0.01 ^A^	1.66 ± 0.01 ^B^ (11.04 ± 0.07 ^E^)
5	Kynurenic acid	247.43 ± 0.09 ^G^	220.25 ± 0.05 ^H^	27.18 ± 0.06 ^H^ (12.34 ± 0.03 ^F^)
6	Caffeic acid	480.05 ± 0.01 ^I^	437.42 ± 0.01 ^I^	42.63 ± 0.01 ^I^ (9.75 ± 0.00 ^D^)
7	Coumaric acid	19.09 ± 0.02 ^B^	18.15 ± 0.01 ^B^	0.94 ± 0.01 ^A^ (5.17 ± 0.08 ^A^)
8	Lutein	59.22 ± 0.08 ^E^	55.60 ± 0.02 ^E^	3.62 ± 0.10 ^D^ (6.52 ± 0.18 ^B^)
9	Luteolin	84.30 ± 0.06 ^F^	78.73 ± 0.02 ^F^	5.57 ± 0.07 ^F^ (7.08 ± 0.09 ^C^)

All values are mean ± SD (*n* = 3). ^A–I^ Means with different superscripts in the same column are significantly different at *p* < 0.05 by Duncan’s multiple range tests.

## Data Availability

Not applicable.
